# CT imaging indications correlate with the degree of lung adenocarcinoma infiltration

**DOI:** 10.3389/fonc.2023.1108758

**Published:** 2023-03-10

**Authors:** Wenchen He, Gang Guo, Xiaoxiang Du, Shiping Guo, Xiaofei Zhuang

**Affiliations:** ^1^ Cancer Hospital Affiliated to Shanxi Medical University/Shanxi Province Cancer Hospital/ Shanxi Hospital Affiliated to Cancer Hospital, Chinese Academy of Medical Sciences, Taiyuan, Shanxi, China; ^2^ Shanxi Province Cancer Hospital/ Shanxi Hospital Affiliated to Cancer Hospital, Chinese Academy of Medical Sciences/Cancer Hospital Affiliated to Shanxi Medical University, Taiyuan, Shanxi, China; ^3^ Department of Cardiothoracic Surgery, Lvliang People's Hospital, Lvliang, Shanxi, China

**Keywords:** lung adenocarcinoma, ground glass nodule, CT, imaging group characteristics, pathology, logistic regression analysis

## Abstract

**Background:**

Ground glass nodules (GGN) of the lung may be a precursor of lung cancer and have received increasing attention in recent years with the popularity of low-dose high-resolution computed tomography (CT). Many studies have discussed imaging features that suggest the benignity or malignancy of GGN, but the extent of its postoperative pathological infiltration is poorly understood. In this study, we identified CT imaging features that indicate the extent of GGN pathological infiltration.

**Methods:**

A retrospective analysis of 189 patients with pulmonary GGN from January 2020 to December 2021 at Shanxi Cancer Hospital was performed. Patients were classified according to their pathological type into non-invasive adenocarcinoma [atypical adenomatous hyperplasia (AAH) and adenocarcinoma *in situ* (AIS) in a total of 34 cases], micro-invasive adenocarcinoma (MIA) in 80 cases, and invasive adenocarcinoma (IAC) in a total of 75 cases. The general demographic data, nodule size, nodule area, solid component, CT indications and pathological findings of the three groups of patients were analyzed to predict the correlation between GGN and the degree of lung adenocarcinoma infiltration.

**Results:**

No statistically significant differences were found among the three groups in general information, vascular signs, and vacuolar signs (P > 0.05). Statistically significant differences among the three groups were found in nodule size, nodule area, lobar signs, pleural traction, burr signs, bronchial signs, and solid components (P < 0.05). Logistic regression equation tests based on the statistically significant indicators showed that nodal area, lobar sign, pleural pull, burr sign, bronchial sign, and solid component were independent predictors of lung adenocarcinoma infiltration. The subject operating characteristic (ROC) curve analysis showed that nodal area is valuable in predicting GGN infiltration.

**Conclusion:**

CT-based imaging indications are useful predictors of infiltrative adenocarcinoma manifested as pulmonary ground glass nodules.

## Introduction

Lung cancer is one of the most common cancers in the world and the most common cause of cancer death. The proportion of adenocarcinoma in lung cancer has exceeded that of squamous cancer (1). In China, the mortality rate of lung cancer has always been the highest (2). With the improvement in people’s health awareness and the popularity of low-dose high-resolution CT, more and more small lung nodules are being screened. Lung nodules are one of the early symptoms of lung cancer. Early detection, diagnosis, and treatment can effectively reduce the mortality rate of lung cancer. Recently, a population-based randomized controlled trial showed that low-dose CT screening was associated with a lower lung cancer mortality rate than no screening in high-risk patients (3). The pathologic staging of small adenocarcinomas of the lung, which contained a large number of GGN, was classified as Noguchi type A, B, and C (4). Early researchers who found ground glass nodules advocated surgical resection. During follow-up, however, it was found that most patients with ground glass nodules had a long survival with minimal recurrence and metastasis. Researchers therefore began to reflect on whether ground glass nodules could be monitored first after discovery, and only operated on when necessary (5). A pulmonary ground glass nodule is defined as a ground glass-like change that surrounds the lung parenchyma with a mildly hyperintense shadow (6). Depending on the presence or absence of a solid component in the nodule, it can be classified into three categories: pure ground glass nodules (PGGN), heterogeneous ground glass nodules (HGGN), and true solid component ground glass nodules (RGGN) (7). PGGN is prone to develop into focal interstitial fibrosis, AAH, or AIS, whereas HGGN and RGGN are more likely to be malignant than PGGN, typically end up becoming MIA or IAC (8, 9). The solid component is generally an indicator of aggressiveness, so the greater the proportion of the solid component, the worse the prognosis (10). In 2011, the International Association for the Study of Lung Cancer, the American Thoracic Society, and the European Respiratory Society proposed AIS—a small adenocarcinoma with limited size, lacking interstitial, vascular, or pleural infiltration, and has a survival rate of nearly 100% after complete resection. It was also recognized as the second type of preinvasive lung adenocarcinoma in addition to AAH (11). In 2015, AIS was added as a pre-invasive lesion to the WHO classification of lung tumors, and a diagnostic criteria for AIS were proposed (12). The 2021 WHO lung tumor classification also include adenocarcinoma *in situ*, which were previously part of adenocarcinoma of the lung, as pre-invasive lesions, or non-invasive carcinoma (13). Through imaging data and the results of previous studies, clinicians can roughly determine the benignity or malignancy of nodules, but no consensus has been reached on how to reliably identify the extent of lung adenocarcinoma infiltration. Accurate differentiation between non-invasive and infiltrative lesions is crucial for treatment planning and can greatly reduce overtreatment of lung nodules. The new classification of lung adenocarcinoma proposes a suitable classification method for the first time, which improves the 5-year survival rate for complete surgical resection of AAH/AIS and MIA to 100%, and the treatment approach and prognosis prediction of IAC become more reasonable (14, 15). Preoperative predictive CT image indications for pathological staging are of great value in guiding clinical treatment, and correct diagnostic results can effectively improve patient survival. In this study, we analyzed CT imaging indications of ground glass nodules with surgically resected, pathologically confirmed lung adenocarcinoma to explore the correlation between them and to provide a reference basis for clinicians to identify the degree of GNN infiltration.

## Methods

### Clinical information

One hundred and eighty-nine patients with GGN confirmed by surgical pathology in the Department of Thoracic Surgery II of Shanxi Cancer Hospital from January 2020 to December 2021 were selected with the following inclusion criteria:(1) No preoperative puncture and related antitumor treatment. (2) Patients were ≥18 years old. (3) Chest CT showed pulmonary ground glass nodules. (4) Postoperative pathological findings of AAH, AIS, MIA, or IAC. (5) Neck ultrasound, abdominal ultrasound, bone scan, and cranial MRI did not reveal distant metastases. The exclusion criteria are: (1) Patients with incomplete basic clinical information. (2) Patients with other concurrent malignant tumors. (3) Patients with multiple foci in both lungs. (4) Patients with incomplete imaging data. Among the 189 selected patients, 66 were males and 123 were females. 34 were diagnosed with AAH+AIS, 80 with MIA and 75 with IAC. Lung segmental resection, lung wedge resection, or lobectomy was performed according to intraoperative freezing results.

### CT imaging

Thoracic CT in Shanxi Cancer Hospital (①GE Discovery CT750 HD 64-row spiral CT) was used with a tube voltage of 100kv, a tube current of 450ma (sometimes automatically adjusted), a pitch of 1.375:1, a layer thickness of 5.0mm, a field of view of large Body (adjustable), and an image matrix of 512x. ②Enhancers iodixanol/iopromide were injected through the median elbow vein, with an intravenous flow rate of 3.5ml/s. The scans were performed from the lung apex to the lung base, with patients taking a deep inhalation and then holding the breath to start scanning. All parameters were obtained from chest CT flattening + enhancement.

CT images were analyzed by two senior attending physicians. When the conclusions were inconsistent, agreements were reached after department-wide discussions. Observations of interest included: ①Nodule location: left upper lung lobe, left lower lung lobe, right upper lung lobe, right middle lung lobe, or right lower lung lobe. ②Density: pure ground glass nodules or mixed ground glass nodules. ③Nodule size: the maximum diameter of a nodule was measured through the cross-section of the image. ④Nodule area: calculated by taking the square of the measured nodule size. ⑤Nodule nature: whether there are features such as burr, lobulation, vacuolation, vascular shadow, bronchial sign, pleural traction, and other characteristics.

### Pathological diagnosis

189 GGN specimens were surgically resected. The specimens were fixed by 4% formaldehyde, sectioned, paraffin embedded, filmed, routine hematoxylin-eosin stained (HE staining), and some were further analyzed by immunohistochemistry to clarify the tumor staging. Two senior pathologists worked together to make diagnosis. When the results were inconsistent, consensuses were reached through whole-department discussions. The diagnostic criteria are listed as following: (1) AAH: focal lesion (≤0.5 cm) with mild to moderate epithelial atypical hyperplasia, growing along the alveolar or respiratory bronchial wall without interstitial inflammatory reaction and fibroplasia; (2) AIS: focal lesion (≤3.0 cm) with epithelial growth along the alveolar wall without interstitial, vascular, or pleural infiltration; (3) MIA: focal lesion (≤3.0 cm) with alveolar cells growing mainly in an adnexal pattern and infiltrating foci ≤0.5 cm; (4) IAC: focal lesion (≤3.0 cm) with infiltrating lesions >0.5 cm.

### Observed indicators

A one-way analysis was performed by comparing the general data and CT indications of the three groups. The indications with predictive power were then included in the logistic regression analysis for multifactor analysis and subject work curves.

### Statistical analysis

All data in this study were statistically analyzed using SPSS 25.0 statistical software. Firstly, a normality test was performed. The measurement data obeying normal distribution were expressed as mean ± standard deviation, and t-test for independent samples was used for comparison between groups. The measurement data of skewed distribution were expressed as median (interquartile spacing), and rank sum test was used for comparison between groups. Count data were expressed as cases and rates, and comparisons between groups were made using the χ^2^test or Fisher’s exact probability method, and trendχ^2^ test plots were drawn. The variables were first subjected to univariate analysis, and the variables with statistically significant differences in results were included in multivariate multiple logistic regression analysis to analyze independent risk factors for infiltrative pulmonary nodules. Subject operating characteristic (ROC) curves were plotted to calculate the area under curve (AUC), to determine the diagnostic cutoff values, and to analyze the sensitivity and specificity of the model. In this study, the diagnostic value of AUC was considered low between 0.5 and 0.7, moderate between 0.7 and 0.9, and high when > 0.9. All data with P ≤ 0.05 were considered statistically significant.

## Results

A total of 189 patients were included in the study, 34 with AAH+AIS, 80 with MIA, and 75 with IAC. The general information and CT indications of the patients are shown in **Table 1**.

**Table 1:** Basic characteristics of patients with different pathological types.

Patient Characteristics	AAH+AIS(n=34)	MIA(n=80)	IAC (n=75)	P value
Age (years)	57.5( ± 7.874)	61.5( ± 8.779)	59.0 ( ± 9.892)	0.092
Male	13(38,2%)	24(30.0%)	29 (38.7%)	0.509
History of smoking	9 (26.5%)	18 (22.5%)	20 (26.4%)	0.858
Height (cm)	163.2( ± 9.361)	161.4( ± 7.466)	163.5 ( ± 7.724)	0.104
Body weight (kg)	60.5( ± 11.169)	62.0( ± 10.736)	62.0 ( ± 11.144)	0.842
BMI (kg/m^2^)	23.1( ± 3.037)	24.1( ± 3.766)	23.3( ± 3.126)	0.893
Nodule area (cm^2^)	0.9( ± 0.550)	1.43( ± 1.712)	2.2( ± 2.712)	<0.001
Nodule characteristics				
with lobed sign	1(2.9%)	24(30.0%)	25 (33.3%)	0.002
With vascular shadow	12 (35.3%)	24(30.0%)	21(28.0%)	0.762
Empty bubble sign	2(5.9%)	11 (13.8%)	15(20.0%)	0.157
With pleural pull	3(8.8%)	22 (27.5%)	30(40.0%)	0.004
Burr sign	1(2.9%)	17 (21.3%)	23 (30.7%)	0.004
With bronchial signs	1(2.9%)	20(25.0%)	23 (30.7%)	0.006
With solid components	3(8.8%)	23 (28.7%)	40 (53.3%)	<0.001

**Figure 1:**
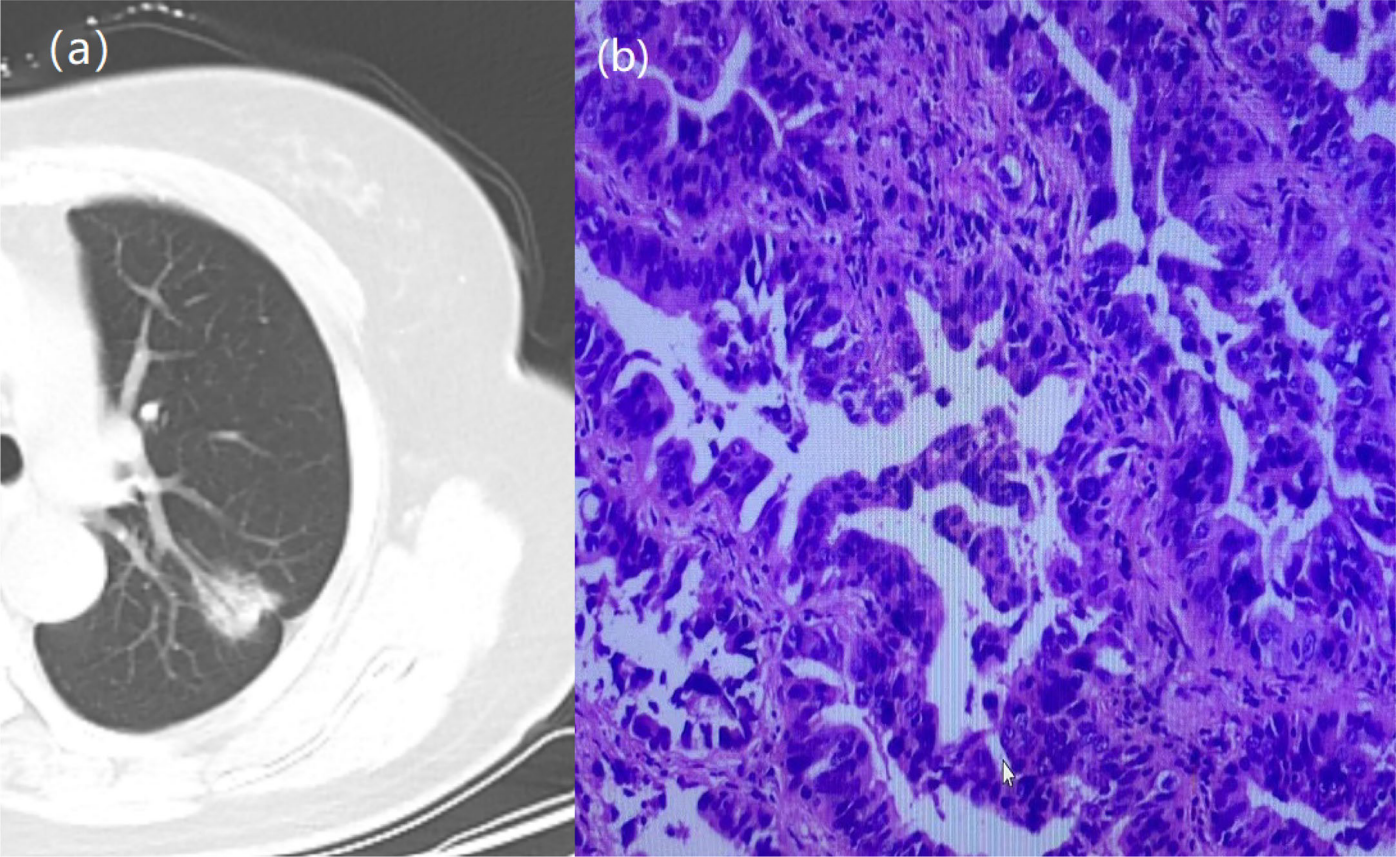
A 64-year-old woman with a GGN. CT showed lobulation, pleural traction, burr, bronchial signs. **(A)** Chest scan. **(B)** pathologically confirmed for IAC.

**Figure 2:**
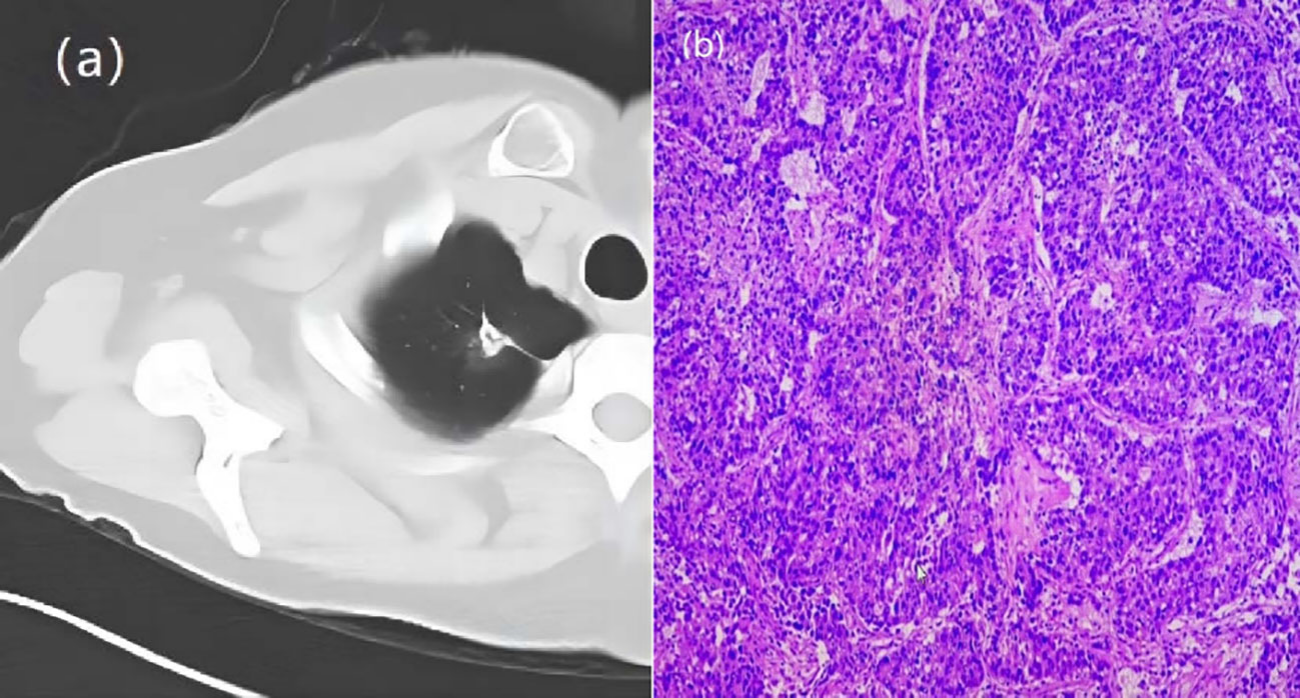
A 66-year-old woman with a GGN. CT showed lobulation, pleural traction, burr. **(A)** Chest scan. **(B)** pathologically confirmed for IAC.

**Figure 3:**
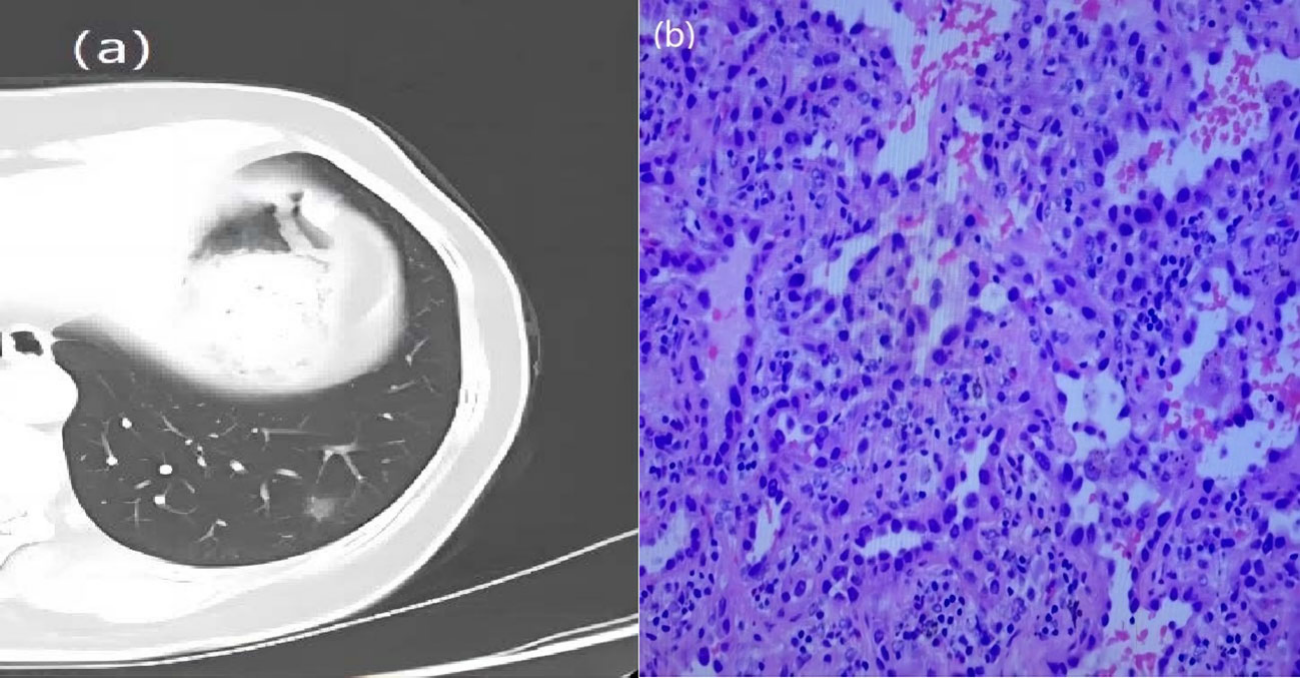
A 57-year-old woman with a GGN. CT showed lobulation and burr. **(A)** Chest scan. **(B)** pathologically confirmed for MIA.

**Figure 4:**
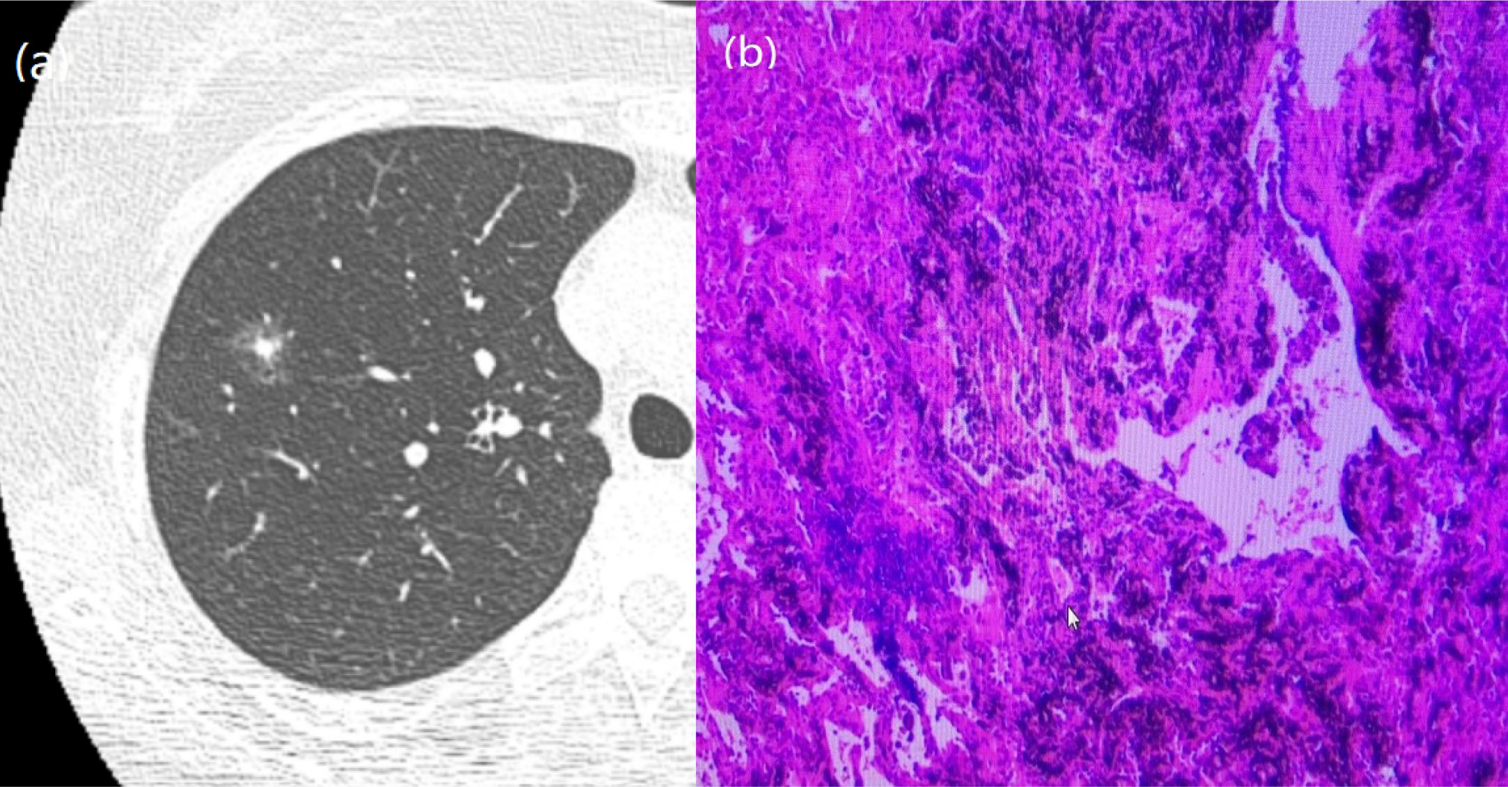
A 51-year-old man with a GGN. CT showed burr. **(A)** Chest scan. **(B)** pathologically confirmed for AIS.

**Figure 5:**
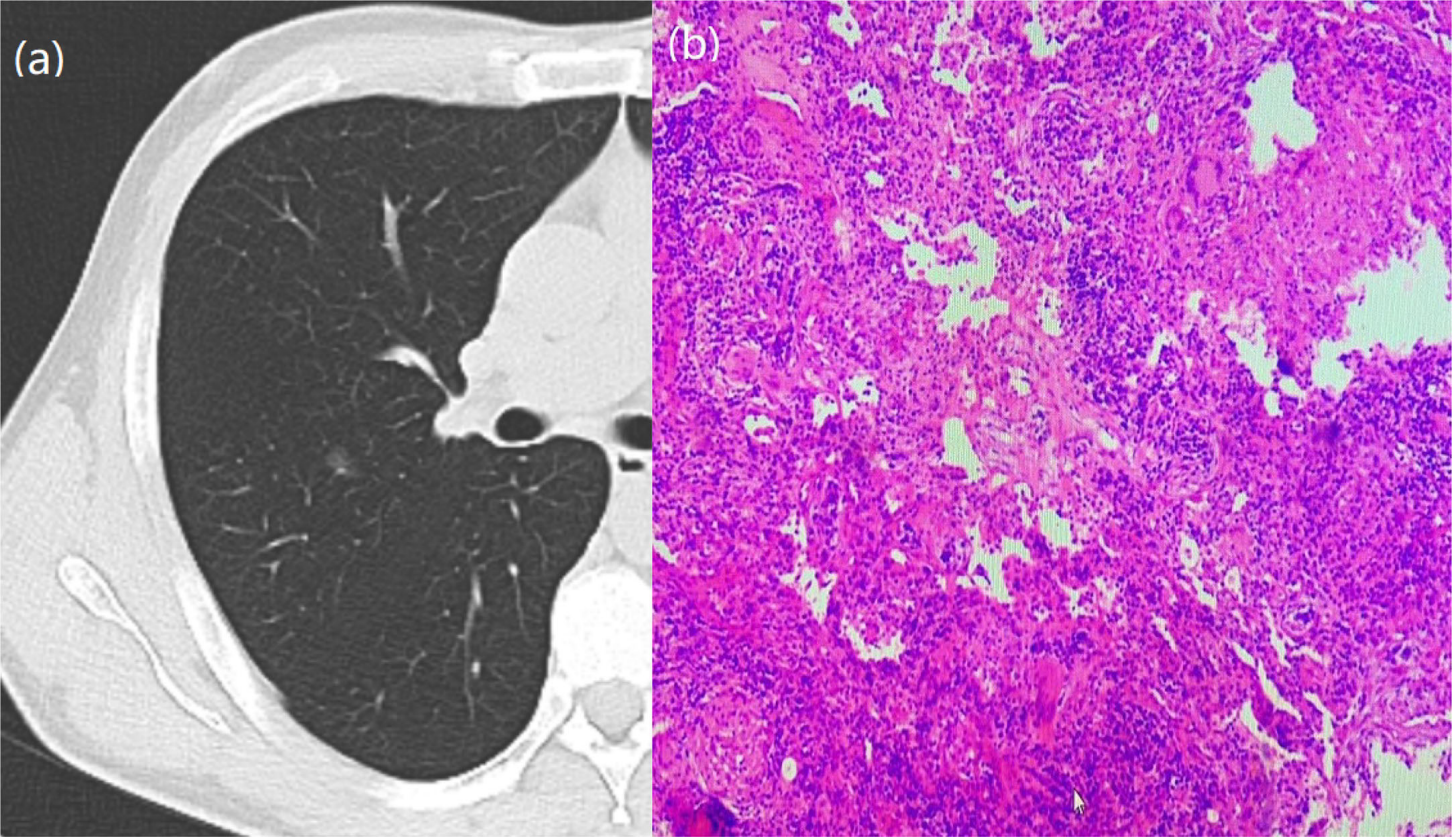
A 63-year-old woman with a GGN. **(A)** Chest scan. **(B)** pathologically confirmed for AAH.

By univariate analysis, there were no statistically significant differences in gender, age, history of smoking, height, weight, BMI, vascular sign (P=0.762), and vacuolar sign (P=0.157) between AAH/AIS, MIA, and IAC patients. However, nodule size, nodule area, lobar sign, pleural traction, burr sign, bronchial sign, and solid component were all different with statistical significance among the three groups. From AAH/AIS to MIA to IAC, the probability of nodule size, nodule area, lobar sign, pleural traction, burr sign, bronchial sign, and solid component gradually increased with increasing infiltration (**Figures 1**–**5**).

Multi-factor analysis was performed by binary logistic regression (**Table 2**). The effect of nodal area growth on the occurrence of MIA (b=0.747, OR=2.110, 95% CI 1.052 - 4.233, P=0.036) and IAC (b=0.968, OR=2.634, 95% CI 1.301 - 5.333, P=0.007) in nodal pathology was statistically significant. As the nodal area grew, nodules were more inclined to develop MIA and IAC than AAH+AIS with OR>1. Growth in the nodal area is a risk factor for the development of MIA and IAC in nodules.

**Table 2:** Multiple logistic regression analysis of each CT sign and pathology type.

	Variables	B-value	SE value of B value	Wald value	P-value	OR value	OR value 95% CI
MIA	intercept distance	9.023	2.231	16.358	0.000052		
	Nodule size	0.747	0.355	4.418	0.036	2.110	1.052 - 4.233
	Lobing sign = none	-2.776	1.085	6.551	0.010	0.062	0.007 - 0.522
	Lobular sign = yes	0^b^	–	–	–	–	–
	Pleural pull = none	-1.470	1.103	4.123	0.042	0.230	0.056 - 0.950
	Pleural traction = yes	0^b^	–	–	–	–	–
	Burr sign = no	-2.379	1.103	4.653	0.031	0.093	0.011 - 0.681
	Burr sign = Yes	0^b^	–	–	–	–	–
	Bronchial signs = none	-2.505	1.082	5.359	0.021	0.082	0.010 - 0.681
	Bronchial signs = yes	0^b^	–	–	–	–	–
	Solidity = None	-1.456	0.709	4.215	0.040	0.233	0.058 - 0.936
	Solidity = Yes	0^b^	–	–	–	–	–
IAC	intercept distance	10.028	2.282	19.313	0.000011		
	Nodule size	0.968	0.360	7.237	0.007	2,634	1.301 - 5.333
	Lobing sign = none	-2.724	1.119	5.925	0.015	0.066	0.007 - 0.588
	Lobular sign = yes	0^b^	–	–	–	–	–
	Pleural pull = none	-2.047	0.755	7.362	0.007	0.129	0.029 - 0.566
	Pleural traction = yes	0^b^	–	–	–	–	–
	Burr sign = no	-3.075	1.124	7.476	0.006	0.046	0.005 - 0.419
	Burr sign = Yes	0^b^	–	–	–	–	–
	Bronchial signs = none	-2.734	1.107	6.095	0.014	0.065	0.007 - 0.569
	Bronchial signs = yes	0^b^	–	–	–	–	–
	Solidity = None	-2.435	0.729	11.172	0.001	0.088	0.021 - 0.365
	Solidity = Yes	0^b^	–	–	–	–	–

The presence of lobulated signs had a statistically significant effect on the nodal pathogenesis of MIA (b=-2.776, OR=0.062, 95% CI 0.007 - 0.522, P=0.010) and IAC (b=-2.724, OR=0.066, 95% CI 0.007 - 0.588, P=0.015). Compared with the nodules with lobulated signs, the nodules without lobulated signs were more likely to have AAH +AIS than MIA or IAC, with OR < 1. The absence of lobulated signs was a protective factor against the occurrence of MIA and IAC in nodules.

The presence of pleural traction had a statistically significant effect on the nodal pathogenesis of MIA (b=-1.470, OR=0.230, 95% CI 0.056 - 0.950, P=0.042) and IAC (b=-2.047, OR=0.129, 95% CI 0.029 - 0.566, P=0.007). Compared with nodules with pleural traction, the nodules without pleural traction were more likely to develop AAH+AIS than MIA or IAC, with OR < 1. The absence of pleural traction was a protective factor against the development of MIA and IAC in nodules.

The presence of burr sign had a statistically significant effect on the nodal pathogenesis for MIA (b=-2.379, OR=0.093, 95% CI 0.011 - 0.681, P=0.031) and IAC (b=-3.075, OR=0.046, 95% CI 0.005 - 0.419, P=0.006). Compared with the nodules with burr sign, the nodules without burr sign were more likely to have AAH+AIS than MIA or IAC, with OR < 1. Burr-free signs were a protective factor against the occurrence of MIA and IAC in nodules.

The presence of bronchial signs has a statistically significant effect on the nodal pathogenesis of MIA (b=-2.505, OR=0.082, 95% CI 0.010 - 0.681, P=0.021) and IAC (b=-2.734, OR=0.065, 95% CI 0.007 - 0.569, P=0.014) in nodal pathology. Compared with the nodules with bronchial signs, the nodules without bronchial signs were more inclined to develop AAH+AIS than MIA or IAC, with OR < 1. The absence of bronchial signs was a protective factor against the development of MIA and IAC in nodules.

The presence of a solid component has a statistically significant effect on the nodal pathogenesis of MIA (b=-1.456, OR=0.233, 95% CI 0.058 - 0.936, P=0.040) and IAC (b=-2.435, OR=0.088, 95% CI 0.021 - 0.365, P=0.001). Compared with the nodules with a solid component, the nodules without a solid component was more likely to develop AAH+AIS than MIA and IAC, with OR<1. The absence of a solid component was a protective factor against the occurrence of MIA and IAC in nodules.

**Figure 6:**
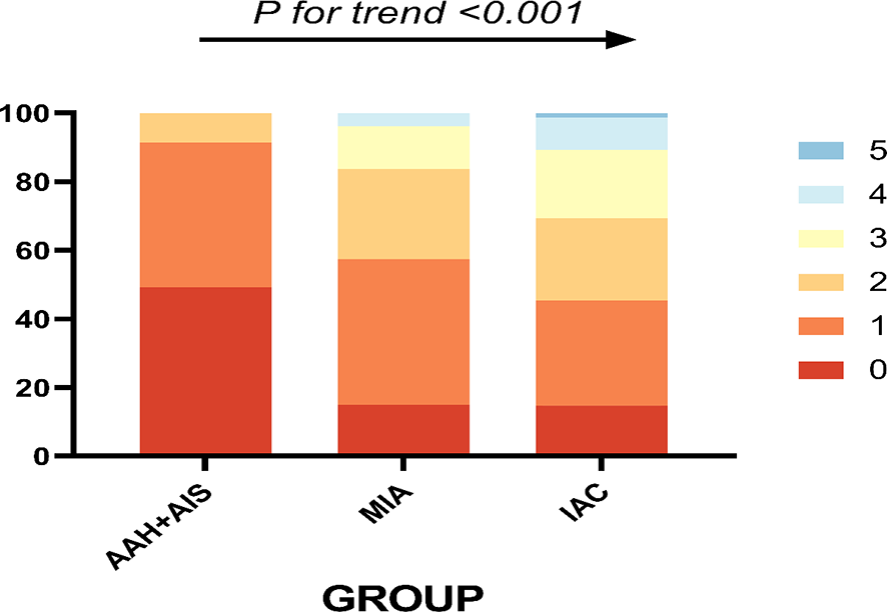
The different pathological types and their corresponding number of abnormal CT signs, 0-5 indicates the number of CT signs.

The trend chi-square test for the difference in the number of CT signs in different pathological types showed that the number of statistically significant CT signs increased with the depth of tumor infiltration (**Figure 6**).

**Figure 7:**
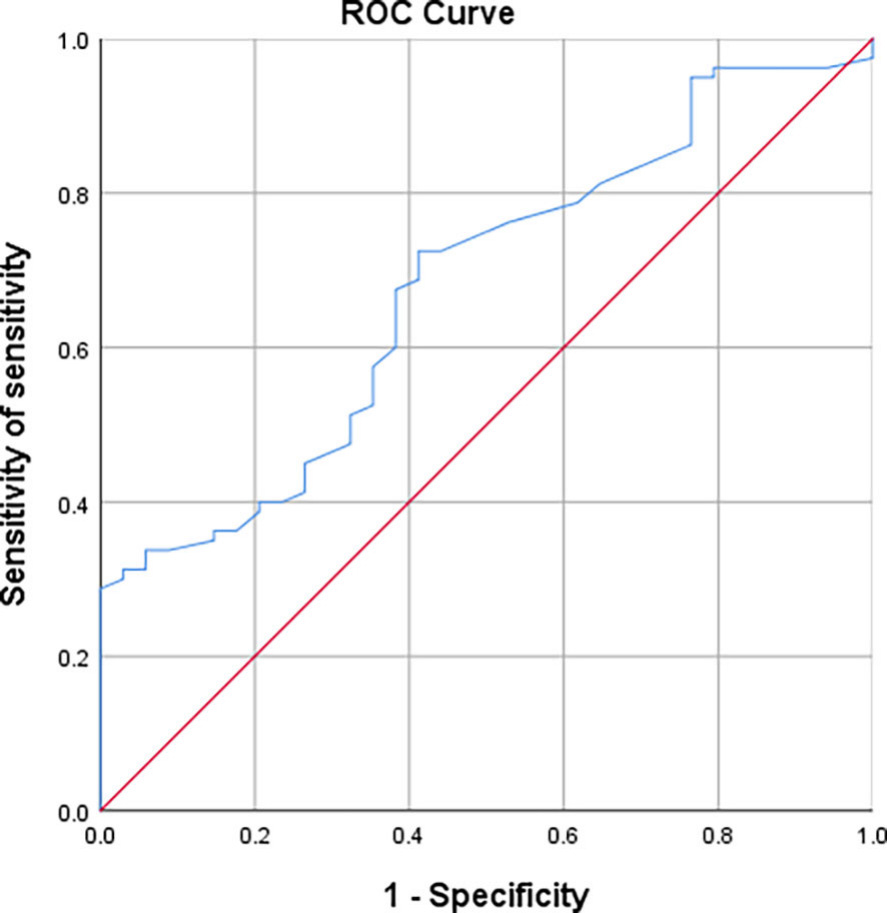
Working curves of subjects with nodal area indications differentiating AAH/AIS and MIA.

ROC curves were plotted between the two groups of AAH/AIS and MIA regarding nodal area. The results showed that nodal area is a useful indicator for the differential diagnosis of AAH+AIS and MIA [AUC (95% CI) = 0.677 (0.574-0.780)] with an optimal cut-off value of 0.975 cm^2^, which corresponds to a sensitivity and specificity of 72.5% and 58.8%, respectively (**Figure 7**).

**Figure 8:**
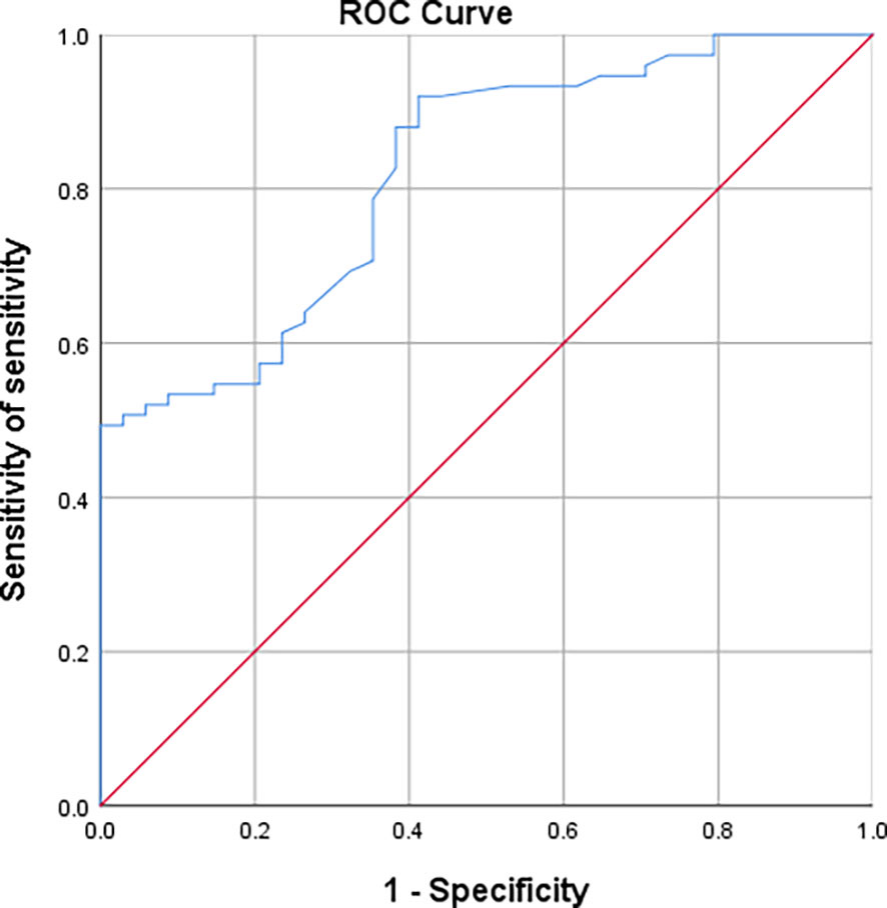
Working curves of subjects with nodal area indications differentiating AAH/AIS and IAC.

The ROC curves of nodal area between AAH/AIS and IAC groups showed that nodal area was similarly useful in the differential diagnosis of AAH+AIS and IAC [AUC (95% CI)=0.819 (0.738-0.900)], with an optimal cut-off value of 0.970cm^2^, corresponding to a sensitivity and specificity of 92% and 58.8%, respectively (**Figure 8**).

## Discussion

Adenocarcinoma of the lung is the most important histological subtype of lung cancer. Early diagnosis and clinical intervention are particularly important for patient prognosis. It is generally believed that ground glass nodules smaller than 5mm do not require antibiotics and follow-up visits by patients are sufficient. Ground glass/mixed ground glass nodules newly developed within one year are generally considered inflammatory lesions and are treated with antibiotics first. If no significant change is observed after anti-inflammation treatment, these nodules are considered tumors and are surgically removed. Pulmonary nodules are constantly evolving. As a result, the size, the amount of solid components, and the proportion of various CT indications of these pulmonary nodules are extremely variable, which also corresponds to the gradual transition of AAH/AIS-MIA-IAC in pathological staging. Consequently, CT signs are important indicators to determine the degree of infiltration. In this paper, we established that the higher the proportion of various indications on CT imaging of pulmonary ground glass nodules, the higher the risk of pathological staging from AAH/AIS to MIA to IAC. Additionally, these indications also have high sensitivity and specificity for the diagnosis of infiltration.

Nodule size is a good predictor of benign and malignant lung nodules. Hu et al. (16)found that the probability of malignancy was 56% for nodules ≤10 mm in diameter and 88.7% for nodules ≥10 mm in diameter. Shi et al. (17) investigated 1000 lung nodules and found that 67.5% of lung nodules with a diameter ≤10 mm were benign. The probability dropped to 50% with nodules in between 10-20 mm, and further dropped to 15% with nodules ≥20 mm. The result of this study is consistent with previous findings that suggest the larger a nodule becomes, the more malignant it tend to be, and the greater the probability of it being an invasive carcinoma (18). The Fleischner Society guidelines (19) state that pulmonary nodules ≤5 mm in diameter do not need patient follow-up and are not malignant. Consistent with the guideline, no pulmonary nodules ≤5 mm in this study were found to be microinvasive or invasive carcinomas. As the diameter increases, the likelihood of a nodule being MIA or IAC increases, and a positive correlation between the two were identified. Chung et al. showed that careful evaluation of the morphology and size of pulmonary nodules can greatly improve the identification of malignancy (20). However, the differentiation of the degree of infiltration based on nodule size alone is not sufficient, and more indications are needed.

The malignancy risk of pulmonary nodules is known to have the following order: solid nodules > mixed ground glass nodules (MGGO) > pure ground glass nodules (PGGO). The solid component is defined as the density of the solid component inside the cloudy nodule on the lung window that is higher than the density of the lung parenchyma but preserves the normal bronchial and vascular margins (21). It is generally believed that if a solid component is present, then the nodule is more likely to be malignant and will progress from benign to malignant over time. Chest CT of certain benign diseases, such as neutropenia, eosinophilic pneumonia, tuberculosis, and cytomegalovirus can also show a solid component (20). However, unlike benign diseases, malignant nodules persist during the course of follow-up. In this study, it was found that as the solid component of ground glass nodules increases in size, the likelihood of the nodule being an invasive carcinoma also increases. We therefore concluded that a solid component in the nodule is a risk factor for the development of MIA and IAC.

Marginal features of nodules, especially lobulation and burr, are also found to be helpful in distinguishing benign and malignant nodules, as well as in determining the degree of infiltration. Lobulation occurs due to the fact that tumor cells do not grow at the same rate as normal cells. tumor cells is not uniform, which also implies that tumor cells in nodules grow faster. The phenomenon of malignant lobulation is known to be highly associated with malignancy (22). Benign lobulation is the result of proliferation and scarring of adjacent connective tissues, such as a hamartoma, which is a benign disease and should be carefully evaluated on CT (23, 24). In the present study, the degree of malignant infiltration was higher in ground glass nodules with lobulated signs than in those without lobulated signs, which is consistent with previous studies. Burr is caused by a pro-additive response to obstruction of pulmonary vessels or lymphatic vessels by tumor cells, resulting in radial manifestations into the lung parenchyma (25, 26). Nodules with burrs are much more likely to be malignant than nodules with smooth, well-defined margins, with a study demonstrating that burr has a positive predictive value of up to 90% for malignancy (27). Our study showed that burr was an independent factor in the degree of malignant infiltration of ground glass nodules, which is consistent with previous studies and suggests that lobar and burr signs are predictive features for the degree of lung adenocarcinoma infiltration.

Pleural traction is a linear, convoluted shadow between tumor and the pleura, and is closely related to the degree of lung adenocarcinoma infiltration (28). Ichinose et al. analyzed 191 patients with GGN and found that invasive lung cancer accounted for 12% of PGGO lesions, most of which had pleural traction as an indication (29). In their study of preoperative identification of invasive lung adenocarcinoma in patients with purely ground glass nodules using columnar maps, She et al. showed that pleural traction was a predictor of the degree of lung adenocarcinoma infiltration (30). In the present study, we found that pleural traction was more likely to be present in the infiltrating group compared with the anterior lesion group and the microinfiltrate group. Nodules without pleural traction were more likely to develop AAH+AIS, and the absence of pleural traction was a protective factor against the development of MIA and IAC from nodules.

Studies have shown that bronchial signs have predictive value for the degree of infiltration of lung adenocarcinoma and that twisted, dilated bronchi are present in most infiltrating carcinomas (31). In the present study, the abnormal bronchial signs were found to be increased in the presence of infiltration in pulmonary ground-glass nodules, which is consistent with the previous study. When cancer cells infiltrate the bronchi, fibrous hyperplasia is formed, leading to the thickening and stiffening of the bronchial walls, which ultimately produces the abnormal bronchial signs (32). Jin et al. concluded that there is a close relationship between bronchial signs and the tumor aggressiveness determined by pathological histology (33).

Son et al. found that the more lobars, burrs, and solid components appear, the greater the degree of lesion infiltrations (34). Our study has demonstrated that imaging indications such as nodule size, nodule area, solid component, lobar sign, burr sign, pleural pull, and bronchial sign have great significance in differentiating the degree of infiltration of pulmonary ground glass nodules. A multifactorial logistic regression equation test revealed that all the above-mentioned indicators, except for solid component, were independent predictors of lung adenocarcinoma infiltration. Although a single indicator alone is limited in identifying the degree of lung adenocarcinoma infiltration, the reliability of diagnosis can be increased by combining multiple independent indicators. There are still limitations in this study: (1) Only surgical cases were included, which might induce bias. (2) The accuracy of quantitative parameters might be affected by selected parameters of the CT machine. (3) This study is a single center study with a small sample size. A multicenter study with a larger sample size is needed to further validate the conclusions. (4) Shanxi Cancer Hospital is a specialized hospital, with all patients pre-screened by lower level hospitals. Most of the patients have satisfied surgical requirements and most of the postoperative pathology results are microinvasive or invasive carcinoma. The number of patients with atypical adenomatous hyperplasia and carcinoma *in situ* is relatively small.

## Conclusion

In conclusion, CT indications of ground glass nodules are suggestive for identifying postoperative pathologic staging. Comprehensive analysis of nodule size, nodule area, solid component, and CT indications can effectively improve the diagnosis of lung adenocarcinoma. Preoperative predictive CT manifestations of pathological staging can improve preoperative diagnosis and differential diagnosis. Correct diagnostic findings are effective in improving patient survival. Prospective clinical trials of more cases of ground glass nodules are needed to further evaluate and validate the diagnostic value of the factors identified in this study.

## Data availability statement

The raw data supporting the conclusions of this article will be made available by the authors, without undue reservation.

## Ethics statement

The studies involving human participants were reviewed and approved by Shanxi Province Cancer Hospital. Written informed consent for participation was not required for this study in accordance with the national legislation and the institutional requirements.

## Author contributions

WCH, SPG and XFZ conceived and designed the study and helped to draft the manuscript. XXD performed the data collection. GG performed the statistical analysis. All authors read and critically revised the manuscript for intellectual content and approved the final manuscript.
